# Challenges Faced by Healthcare Professionals in Screening Newborns for Congenital Heart Defects in Pakistan

**DOI:** 10.3390/ijns11040095

**Published:** 2025-10-15

**Authors:** Ijaz ul Haq, Muhammad Imran Khan, Amir Muhammad, Majid Ali, Xiaojing Hu, Guo-Ying Huang

**Affiliations:** 1 National Management Office of Neonatal Screening Project for CHD, Children’s Hospital of Fudan University, National Children’s Medical Center, Shanghai 201102, China; 2 Department of Public Health & Nutrition, The University of Haripur, Haripur 22620, Khyber Pakhtunkhwa, Pakistan; 3 Department of Clinical Nutrition, College of Applied Medical Sciences, King Faisal University, Al-Ahsa 31982, Saudi Arabia; 4 Saidu Group of Teaching Hospitals, Saidu Sharif, Swat 19200, Khyber Pakhtunkhwa, Pakistan; 5 Pediatrics Department, Lady Reading Hospital, MTI, Peshawar 25000, Khyber Pukhtunkhawa, Pakistan; 6 Department of Public Health, Rawalpindi Medical University, Rawalpindi 46000, Punjab, Pakistan; 7 Shanghai Key Laboratory of Birth Defects, Shanghai 200032, China; 8 Fujian Provincial Key Laboratory of Neonatal Diseases, Xiamen 361006, China

**Keywords:** congenital heart disease, screening, neonate, health systems barriers, challenges

## Abstract

Early and timely screening for congenital heart disease (CHD) is one of the key challenges for healthcare professionals (HPs). This study aimed to identify barriers to the screening of CHD among healthcare professionals in Khyber Pakhtunkhwa, Pakistan. A qualitative cross-sectional study was conducted among HPs working in public and private hospitals, and data were analyzed thematically using NVivo 10.0 software until saturation following Braun and Clarke’s framework. Data were reported according to the Standards for Reporting Qualitative Research (SRQR). Participants reported critical gaps in CHD screening, including scarce resources such as a lack of pulse oximeters and echocardiography machines, inadequate training, and overburdened staff struggling with high patient volumes. Emotional distress was common when diagnosing severe CHDs, compounded by parental reluctance due to low awareness and socioeconomic barriers, including costs and travel distances. Operational inefficiencies, such as inconsistent protocols, weak referral systems, and paper-based record-keeping, further delayed diagnoses. Despite these challenges, HPs emphasized the potential of standardized screening tools, interdisciplinary coordination, and community education to improve detection rates. CHD screening in Pakistan is impeded by resource limitations, systemic fragmentation, and sociocultural factors. Prioritizing equipment procurement, HP training, public awareness campaigns, and policy-mandated screening protocols could enhance early detection.

## 1. Introduction

CHD are among the most common birth defects worldwide, affecting approximately 1.35 million children annually, with a prevalence of 1% [1]. These defects arise during fetal growth and can vary from mild, self-resolving conditions to severe malformations, also known as critical CHD, which accounts for about one-fourth of CHD cases and requires immediate medical intervention. Delays in detecting such cases can lead to cardiovascular compromise and further organ dysfunction. Early detection and management reduce the risk of infant morbidity, mortality, and related disabilities [2]. High-income countries have implemented various screening methods to minimize delays in CHD detection, whereas low- and middle-income countries (LMICs) lack standardized procedures on a wider scale. In many parts of the world, early detection is often delayed, leading to complications such as impaired growth and development, reduced quality of life (QOL), and decreased survival rates [3,4]. Therefore, screening for CHD in newborns is an essential component of neonatal care. However, despite its importance, healthcare professionals (HPs) face numerous challenges in conducting effective screening.

The screening process varies across regions. It typically involves a physical examination, cardiac auscultation, pulse oximetry, and sometimes echocardiography (Echo) [5]. Fetal ultrasound is a key component of CHD screening in high-income countries, where more than 50% of cases are detected before birth [6]. However, in many LMICs, including Pakistan, such services are not widely available due to a lack of equipment and trained personnel. Observing newborns for signs such as bluish skin (cyanosis), abnormal heart sounds (murmurs), and breathing difficulties aids in identifying CHD [7].

There are numerous challenges in the early screening of newborns, and a significant portion of the population in low- and middle-income countries (LMICs) lacks access to standard fetal CHD detection procedures, resulting in diagnostic delays [8]. Clinical examination alone is often insufficient, as newborns may remain asymptomatic until the closure of the ductus arteriosus and foramen ovale, completing the transition from fetal to neonatal circulation. Cyanosis may not manifest until the oxygen saturation (SpO_2_) drops below 80%. Murmurs, despite being a significant clinical indicator of CHD, may be absent in specific critical instances or may be misleading owing to anatomical variations, the degree of pulmonary vascular resistance, or impaired ventricular function. Furthermore, the detection of complex CHDs and subtle abnormalities persists as a challenge when relying exclusively on routine clinical examinations [9]. Pulse oximetry (POX) is a reliable, non-invasive, and easy-to-use tool for detecting critical CHDs and is widely used for screening [10]. However, limitations such as false positives or negatives due to poor technique, calibration issues, variations in accuracy at high altitudes, skin pigmentation differences, or environmental conditions can lead to significant challenges in effective screening. Professor Guoying Huang’s research group has developed a novel “dual index” screening method that integrates pulse oximetry with cardiac murmur auscultation for the early detection of CHD in neonates within 6 to 72 h after birth. So, internationally, there are POX and dual-index screening methods that have been developed [11]. The “dual-index” has not yet been utilized in Pakistan. In Pakistan, a combination of clinical examination and pulse oximetry (POX) is employed for early screening of CHD in newborns. However, are there any challenges faced in real-world activities when conducting these screenings? We reviewed several references to identify the challenges reported by healthcare professionals involved in newborn CHD screening. These include a lack of specialized equipment, inadequate training, insufficient follow-up care, limited institutional support, poor healthcare infrastructure, low family awareness, and adherence to essential care [12]. Similarly, HPs working in low- and middle-income countries such as Pakistan have highlighted the burden of heavy workloads, insufficient resources, difficulties in accessing screening equipment [13] and challenges in obtaining ECHO services. They also face issues with transportation, a high volume of home deliveries, and the absence of health records for newborns, all of which complicate the screening process [14].

The healthcare system in Pakistan comprises both the public and private sectors. With a population exceeding 220 million, the country’s residents seek medical treatment through these institutions. Despite serving a large population, the healthcare system confronts many challenges. These include limited financial resources, a substantial workload on medical practitioners, a dearth of specialist physicians and medical equipment, the emigration of healthcare professionals due to limited opportunities, an uneven distribution of resources among provinces, the absence of public health insurance, and the limited scope of the Sehat Sahulat program [15]. The government initiated the Sehat Sahulat program, under which Sehat Insaf Cards were issued to facilitate the treatment of hospitalized chronic diseases. A specific amount is allocated for each family. In cases where the treatment costs surpass this predefined limit, patients are required to bear the additional expenses. Furthermore, the disruption of the program’s continuity for political reasons has had an adverse impact on its efficacy [15]. According to a study, every year 21,000 operations are performed, and surgeries per million population is 108.8 [16]. The cost of an open-heart procedure is around USD 3500. The cost of an Atrial Septal Defect (ASD) device is around USD 3000. In 2021, in Punjab, around 3000–4000 pediatric cardiac surgical procedures are performed each year. There is a lack of infant and neonatal cardiac facilities in Pakistan, but usually surgeries are performed for more than 1 year [17].

This study aims to explore the perspectives of professionals working in resource-constrained settings and highlight their role in CHD-related outcomes in Khyber Pakhtunkhwa, Pakistan. It examines the limitations of current screening practices, disparities in available resources, and the need for improved screening strategies. Addressing these challenges is crucial for better education, enhanced early detection, and the reduction of CHD-related morbidity and mortality. The study also discusses potential strategies to overcome these challenges and improve outcomes for affected newborns.

## 2. Methodology

### 2.1. Study Design

The study employed a qualitative descriptive research design, utilizing semi-structured interviews to obtain a detailed understanding of the challenges HPs face when screening for CHD in newborns. The study adhered to the Standards for Reporting Qualitative Research (SRQR) [18].

### 2.2. Setting

We selected the Swat District, situated in the central region of the Malakand Division, which houses the sole teaching hospital that serves approximately 10 million people in the area. All HPs were sourced from three facilities that provide early CHD screening services for newborns in both the public and private sectors. These facilities, which offer varying levels of care, include Saidu Group of Teaching Hospital Swat (SGTHS), Shifa Hospital Swat (SHS), and the Swat Institute of Medical Sciences (SIMS).

### 2.3. Participants

To maximize diversity, the study included participants from various levels within the obstetrics, pediatric cardiology outpatient departments, and NICUs of STHS, SHS, and SIMS. Eligible participants had experience in neonatal care and CHD screening, utilizing physical examination, pulse oximetry (POX), or echocardiography. The study included professionals with various titles, including pediatric cardiologists, pediatric cardiac surgeons, pediatricians, cardiologists, medical officers, nursing staff, and allied HPs.

### 2.4. Sample Population

A purposive sampling technique was used to recruit HPs. Initially, HPs were approached to participate voluntarily in interviews. A diverse group of HPs from various levels of the healthcare system was purposively selected to ensure a comprehensive understanding of the challenges and barriers faced across different levels of the healthcare system. They were interviewed exhaustively until no new information was obtained, adhering to the principle of information saturation in qualitative research [19].

### 2.5. Inclusion and Exclusion Criteria

HPs involved in the early screening and diagnosis of CHD in newborns for at least one year were included in the study. Those who were unwilling, had time constraints, or had difficulty in providing detailed answers were excluded.

The interview guide was designed to collect general information about HPs and included 22 primary questions (Supplementary Materials) to explore barriers and challenges. The open-ended questions were developed following an extensive literature review on interview guide development, utilization, and information saturation in qualitative research. A literature review on screening for CHD in newborns and associated challenges was conducted, followed by site visits to gain a comprehensive understanding of the screening process, incorporating insights from experts such as pediatric cardiologists and researchers. The interview guide was refined after several reviews and pilot testing.

### 2.6. Data Collection Procedure

Participants were enrolled and interviewed in March 2025. After obtaining informed consent, interviews were conducted by a researcher with extensive knowledge of bioethics, experience in conducting interviews, and considerable expertise in qualitative research [20]. Twenty-five participants freely elaborated on their responses to open-ended questions designed to understand the challenges during the screening process. They were queried about the screening protocol, its accessibility to the general population, efficiency, limitations, suggestions for improvement, required equipment, its utilization, and constraints. The conduct of ECHO and related constraints was also discussed. Caregiver involvement in screening, along with ethical, cultural, and socioeconomic considerations, was explored. The referral system and its effectiveness for newborns diagnosed with or suspected of having CHD were examined. Follow-up care, its frequency, contributing factors, coordination among facilities and caregivers, the need for specialized training to enhance follow-up care, and suggestions to improve follow-up care were also addressed. The themes that emerged focused on knowledge and attitudes toward CHD screening, perceived barriers to effective screening (e.g., technical, resource-related, or training-related challenges), institutional and systemic factors influencing screening practices, and suggestions for improving CHD detection.

After the initial pilot interviews, the interview guide was refined. Interviews were conducted within the respective departments and private clinics of HPs. In cases where they were unavailable or it was inconvenient for them, interviews were conducted over the phone. Each interview lasted approximately 45 min. Responses were either manually noted or audio-recorded, with the participants’ consent, and transcribed verbatim for analysis. The research team regularly reviewed the data for accuracy during meetings. Data saturation was deemed to have been reached when no new relevant information or data codes emerged. Regular discussions were held on data saturation, transcription, and recurring themes. Data collection was halted once no new insights were obtained during analysis, thus achieving the principle of data saturation.

### 2.7. Ethical Consideration

This study adhered to the Declaration of Helsinki and ethical principles, ensuring respect for cultural norms, confidentiality, and the protection of participants. The ethical committee of the Children’s Hospital of Fudan University, Shanghai, China (No. (2024)/167 approved on 19 August 2024), and the Saidu Group of Teaching Hospital, Swat, Pakistan (No. 47-ERB/SMC/2025 approved on 26 February 2025), approved this study. Participants were informed of the study’s purpose, the voluntary nature of their participation, the option to receive findings upon request, and the confidentiality of data collection and storage. Informed written consent was obtained before conducting the interviews. Following each interview, the findings were shared with participants to ensure the accuracy of their interpretations. Regular discussions among the research team helped to minimize personal biases, and a complete record of the entire process, including interview guides, coding, and theme development, was maintained for transparency.

### 2.8. Data Analysis

Data analyses adhered to the thematic analysis approach outlined by Braun and Clarke (2006) [21]. Patterns and themes were identified through repeated readings of the transcripts and listening to the recordings. After familiarization, initial codes were generated, drawing from both emerging themes within the data and those outlined in the interview guide. Key aspects of the data relevant to the research questions were pinpointed. The codes were subsequently reviewed and refined and then organized into themes that accurately captured the challenges and barriers in CHD screening. The interpretation of the final themes, in conjunction with the literature and theoretical frameworks, culminated in a conclusive statement on the challenges to effective CHD screening.

### 2.9. Methodological Rigor

The Lincoln and Guba criteria were followed to ensure the credibility, dependability, confirmability, and transferability of the data [22]. Engaging participants during interviews and reviewing the study process ensured the rigor, trustworthiness, and credibility of the findings. Similarly, the diverse nature of the participants, selected through purposive sampling, enhances the transferability of the findings. To strengthen the dependability of the findings, the authors/researchers finalized the verbatim transcript after listening to the audio recordings. Bracketing maintained confirmability, avoiding the researcher’s bias and ensuring that the findings followed the participants’ perspectives.

## 3. Results

**Figure 1:**
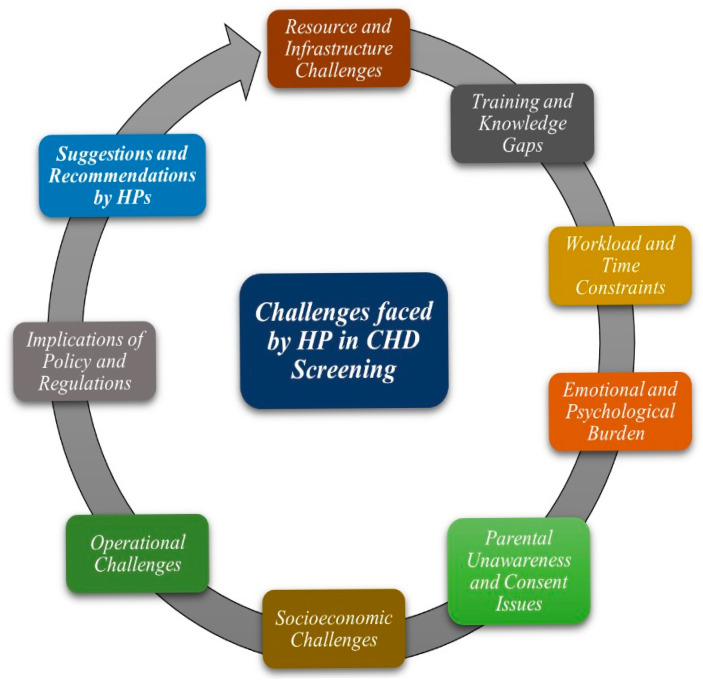
Identified themes of the study.

The current study identified various themes (Figure 1). The demographic characteristics of the study participants are presented in Table 1. A total of 25 HPs were interviewed comprehensively, and data were collected for analysis, excluding the first three interviews, which were conducted as pilot testing. The participants shared their views extensively, which were categorized into several emergent themes, including resource and infrastructure challenges, training and knowledge gaps, workload and time constraints, emotional and psychological burdens, parental unawareness and consent issues, socioeconomic challenges, and operational challenges (Table 2).

### 3.1. Resource and Infrastructure Challenges

Insufficient infrastructure and resources, including pulse oximeters, echocardiogram machines, and trained personnel, were identified as significant obstacles to early screening. Participants emphasized that the scarcity of resources and infrastructure resulted in delays in the early detection and screening of CHD. One participant (P18) remarked, “*There is no dedicated area for screening, and a single pulse oximeter without a replacement in case of malfunction can sometimes disrupt the process.*” Another participant (P24) stated, “*There is limited space for screening newborns and no privacy for caregivers or even us*.”

### 3.2. Training and Knowledge Gaps

Participants reported not receiving adequate training or engaging in practical workshops to aid in the screening processes or to interpret the outcomes. One participant (P23) stated, “*Certainly, I am lacking in training and updated techniques for screening and have requested specialized training*.” Another participant (P9) elaborated, “*I have been screening newborns for congenital heart defects for the past seven years and have not received any training*.” Limited training on CHD screening protocols and a deficiency in updated knowledge impede professional development, resulting in numerous challenges.

### 3.3. Workload and Time Constraints

Due to an overburdened healthcare system and a high birth rate, the limited number of health professionals and equipment leads to challenges in balancing screening with other critical tasks. As one participant (P24) explained,

“*Most of the time, I feel exhausted, and because of the high volume of patients, I cannot allocate sufficient time for individual screenings. At times, we continue our duties for extended periods without any support*.”

### 3.4. Emotional and Psychological Burden

The participants described the diagnosis of severe CHD as a stressful and psychologically burdensome experience for themselves, often leading to emotional reactions. One participant (P20) shared, “*Sometimes, I feel mentally exhausted and need a rest to refresh and calm myself*.” Similarly, (P17) said, “*After the diagnosis of CHD, it is difficult to control emotions. I was in tears on many occasions, feeling for the newborn and the family*.”

### 3.5. Parental Unawareness and Consent Issues

Participants emphasized that the absence of parental awareness and comprehension of the significance of early screening poses a substantial challenge. This frequently results in refusals of consent for additional diagnostic tests and heightens the risk of poorer outcomes. According to one participant (P20), “*Usually, the family is not willing to screen their newborn if there are no obvious features of the disease, and they disregard the guidance provided*.” Parents’ lack of awareness and distrust in healthcare facilities, coupled with a desire to discharge the newborn swiftly, exacerbates this issue. Another participant (P19) described, “*Regrettably, the unawareness within our society complicates matters further, necessitating that we first counsel them on the importance of screening and then on effective management*.”

### 3.6. Socioeconomic Challenges

Participants emphasized various socioeconomic barriers to early screening, including low literacy rates and the inability to cover travel and other costs associated with screening services. One participant (P19) remarked, “*Most of our people lack the resources to access screening, complete the recommended treatment, and adhere to follow-ups. The majority need financial and social support*.” However, no cultural or traditional barriers were reported during the screening process, as described by (P8): “*Fortunately, traditional or cultural practices are not widely observed in healthcare, and I do not consider them to challenge the screening process*.”

### 3.7. Operational Challenges

During the screening procedure, participants identified several operational challenges, including a high volume of patients, coordination issues, a lack of a holistic approach, the absence of a proper referral protocol, interpersonal challenges, variability in screening protocols, and data management issues. Healthcare facilities offering screening services are burdened with high patient numbers and limited time for thorough assessments. Participants reported feeling exhausted, which impacted their performance and the delivery of optimal care, often leading to burnout. Ineffective communication among healthcare professionals and poor coordination with specialists such as obstetricians, pediatricians, and cardiologists impeded comprehensive care. One participant (P5) proposed, “*Improvements could be made by implementing mandatory check-ups for each newborn in the labor room, adopting unified screening criteria, and enhancing coordination among relevant departments to help the improvement of early screening practices*.” Challenges such as insufficient political support, ineffective referral systems, and inadequate connections with specialized care facilities delayed the transfer of cases, resulting in undesirable outcomes.

### 3.8. Implement Policies and Regulations

Participants emphasized that the absence of specific regulations, standardized screening protocols, and follow-up care is a major challenge hindering effective screening. They pointed out that variability in screening practices leads to delayed diagnoses and hampers the timely delivery of life-saving interventions. Screening services are especially scarce in remote areas, necessitating long-distance travel for individuals to reach screening sites. One participant (P2) said, “*We lack a unified screening protocol that could be easily adopted and implemented in remote hospitals*.”

Due to the absence of prenatal or newborn screening using echocardiography during pregnancy or at birth, newborns exhibiting CHD signs must undergo further investigations with echocardiograms and other advanced imaging modalities, such as MRI and CT scans. These services are frequently unavailable on-site, and accessing these facilities for timely examinations presents significant challenges, contributing to high dropout rates. One participant (P14) articulated, “*We are deficient in prenatal services and screening of newborns. The majority of the population struggles to access necessary check-ups or investigations*.” Upon detecting CHD, newborns require immediate attention and early consultation with experts. However, the inadequate healthcare system and the lack of telemedicine services result in delayed diagnosis and appropriate management. Similarly, the absence of an electronic data management system for storage and handling leads to the loss of essential data initially collected on paper forms and stored in registers.

### 3.9. Suggestions and Recommendations from HPs

Based on recommendations from healthcare professionals (HPs), systemic reforms are necessary to improve coronary heart disease (CHD) screening. This includes investing in the provision of basic diagnostic tools, continuous training for HPs, and implementing uniform national standardized screening protocols to increase early detection rates. Additionally, integrating CHD screening into the routine care of newborns, with dedicated facilities and time allocated for comprehensive assessments, is essential. Optimizing workflow to reduce the burden and enhance efficiency is also critical. Establishing clear referral pathways and providing financial support for families will strengthen the referral system and minimize delays in accessing specialized care. Furthermore, awareness campaigns about CHD and the importance of early screening, along with the promotion of interdisciplinary collaboration and team-based training, will improve the coordination of care and enhance early screening practices.

## 4. Discussion

The current study investigated the barriers and challenges that healthcare professionals encounter during the early screening of CHD in newborns. Participants identified multiple obstacles in the screening process, which were categorized into resource- and infrastructure-related challenges, training and knowledge deficiencies, workload and time limitations, emotional and psychological pressures, issues with parental awareness and consent, socioeconomic barriers, and operational difficulties.

The data were collected from healthcare professionals in various roles within the screening process across different hospitals. Clinical examinations are a widely used method for the early screening of CHD in newborns, whereas in some sites, a combination of clinical examinations and POX is employed. The process involves examining for signs of CHD, such as respiratory distress, cyanosis, murmurs, prematurity, and feeding difficulties, and then measuring oxygen saturation using POX. Newborns displaying clinical signs or having oxygen saturation levels below 95% are considered at risk and are advised to undergo echocardiography (Echo) to confirm the presence of CHD. Participants noted that this method is effective in detecting major types of CHD. However, they emphasized that adopting a standardized protocol would enhance the detection of minor defects, leading to earlier diagnosis, a more accurate understanding of the condition, and improved management. Our findings are supported by those of Oster and their team, who conducted their study in 2019 [23]. In our study, participants reported various barriers and challenges encountered during the screening process due to the lack of standardized protocols and specific policies for screening of CHD at birth in Pakistan. These findings were supported by a previous study that stated that limited service availability in remote areas with access challenges and a lack of echocardiograms during antenatal care or at birth lead to delays in diagnosis and essential care provision [24].

The scarcity of equipment, trained personnel, and screening methods poses significant challenges for healthcare providers and patients during the screening process. Limited training opportunities and the absence of practical workshops prevent professionals from enhancing their skills and adopting current techniques, which contributes to delays in the detection of CHD [25]. In some cases, there is no dedicated area for screening, and the absence of Echo machines and operating staff further complicates the process. Additionally, advanced imaging techniques, such as MRI and CT scans, were rarely used.

Participants emphasized several operational challenges that impact the screening process, such as high patient volumes, insufficient coordination, inconsistent screening protocols, inadequate referral systems, and poor data management. In Pakistan, where there is a high birth rate and limited screening services, the healthcare system is overwhelmed, resulting in staff fatigue, time constraints, and insufficient time for individual screenings. Comparable challenges have been documented in previous studies conducted in resource-limited settings [7].

The participants highlighted the absence of coordination among pediatric specialties, an ineffective referral system linking specialized care facilities, and the lack of standardized referral criteria leading to delayed referrals, untimely management, and insufficient follow-up care for monitoring and early detection of complications [26]. The study respondents further elaborated that newborns suspected of having CHD are frequently referred for echocardiograms, but the necessary services are unavailable on-site, causing families to struggle with accessing timely testing, particularly when newborns require oxygen or are in distress. The absence of telemedicine services for early expert consultations further delays diagnosis and treatment, thereby worsening the condition. These findings are in line with the findings reported by Doshi M et al. in 2019 [27]. Currently, screening data are collected on paper prescription sheets and stored in registers. The lack of an electronic data management system for storage and analysis leads to the loss of critical data, making it challenging to monitor trends or determine the factors contributing to CHD.

Standardized follow-up (FU) care, a crucial component in the management of congenital heart disease (CHD), is not universally adopted and is often provided based on the severity of the CHD and the availability of management facilities. Participants highlighted the variability in FU care, as well as other socioeconomic challenges such as lack of awareness, low literacy levels, unaffordability, and limited access to services in remote areas. These factors contribute to delays in early screening and the provision of essential care and management required for newborns with CHD. Similar findings have been suggested by research studies conducted in low- and middle-income countries [28]. No ethical, cultural, or traditional barriers were identified during the screening process. However, the absence of caregiver cooperation and adherence impeded the screening process and the early detection of CHD. According to the participants, many caregivers disregard the essential information provided; they find it difficult to spend time at the hospital and prefer to return home as soon as possible. This makes it challenging to explain and persuade them to seek screening for their child, particularly when there are no apparent signs of CHD. Our findings are supported by the challenges identified by Octavius GS et al. (2023) [25].

The challenges identified by health professionals are similar in many respects and provide a realistic depiction of the situation. They have proposed various suggestions for enhancing the screening process, such as regular training and practical workshops for health professionals involved in CHD screening and management, adopting updated guidelines and standardized protocols, and ensuring access to appropriate equipment, trained personnel, and a dedicated space with online CHD registries and dedicated care coordinators. Services established in remote areas, with standardized follow-up care, could improve CHD outcomes. Some challenges are related to infrastructure, which cannot be changed so easily. Therefore, there is a need for a multi-sectoral approach, involving different stakeholders including the government, NGOs, and charity organizations, to support the infrastructure dedicated to CHD management in low-setting areas of Pakistan. Furthermore, a comprehensive understanding of early CHD screening in newborns requires thorough research. We aim to understand the perspectives of parents and caregivers and evaluate the effectiveness of standardized screening protocols and follow-up mechanisms in the future.

### Strengths and Limitations of the Study

The qualitative design of the study provides in-depth insights and a rich understanding of the individual experiences and perceptions of the barriers and challenges faced by healthcare professionals (HPs) across diverse healthcare settings, thereby enhancing the generalizability of the findings. However, reliance on self-reporting may introduce response bias, as HPs’ answers could be influenced by recall bias, social desirability, or reluctance to disclose challenges related to institutional constraints or a lack of training. Furthermore, the limited time available for interviews due to participants’ heavy workloads could have negatively impacted their ability to engage fully in the interview process.

## 5. Conclusions

This study underscores the various barriers and challenges encountered by healthcare professionals during the screening of congenital heart defects (CHD) in newborns. These include a shortage of equipment, inadequately trained staff, insufficient policies, the absence of a standardized screening protocol, and a lack of coordination among relevant departments. Additionally, the high patient volume, the impoverished socioeconomic conditions of families, and the unawareness or poor compliance of parents or caregivers pose significant obstacles. To ensure the timely detection of CHD in newborns and enhance health outcomes, key challenges related to resources and infrastructure, policy and regulation, operational issues, and socioeconomic support for families must be addressed at both institutional and policy levels.

## Figures and Tables

**Table 1:** Demographic Characteristics of the Participants.

Participant ID	Age	Gender	Job Role	Sector	Locality of Care Provision	Experience in Years
P1	43	M	Cardiologist	Pubic	Urban	6
P2	51	M	Pediatric Cardiologist	Pubic	Urban	4
P3	48	F	Nurse	Pubic	Urban	14
P4	52	M	Cardiologist	Private	Rural	10
P5	41	M	Pediatric Cardiologist	Public	Urban	3
P6	56	M	Cardiologist	Pubic	Urban	12
P7	35	F	Cardiology Technologist *	Private	Urban	7
P8	58	M	Pediatrician	Public	Urban	9
P9	34	F	Nurse	Pubic	Urban	7
P10	50	M	Cardiology Technologist *	Private	Urban	4
P11	42	F	Cardiologist	Pubic	Rural	5
P12	37	M	Medical Officer	Pubic	Urban	2
P13	28	M	Cardiology Technologist *	Pubic	Urban	3
P14	39	M	Interventional Cardiologist	Pubic	Urban	4
P15	35	F	Pediatrician	Private	Urban	5
P16	50	M	Senior Medical officer	Public	Urban	12
P17	26	F	Nurse	Pubic	Urban	2
P18	35	F	Pediatrician	Pubic	Rural	3
P19	48	M	Pediatric Cardiac Surgeon	Private	Urban	4
P20	46	M	Pediatrician	Public	Urban	8
P21	41	F	Woman Medical officer	Public	Urban	2
P22	57	M	Cardiologist	Private	Rural	8
P23	36	M	Cardiology Technologist *	Public	Rural	6
P24	38	F	Woman Medical Officer *	Public	Urban	7
P25	35	M	Pediatrician	Public	Urban	2

* A Cardiology Technologist performs diagnostic tests under the supervision of a cardiologist. * A Medical Officer is a qualified practitioner with an MBBS degree who provides direct patient care. * A Women Medical Officer refers to a female physician with an MBBS qualification, designated to address specific cultural and gender-sensitive healthcare needs.

**Table 2:** Summary of identified sub-themes.

Themes	Sub-Themes
Resource and Infrastructure Challenges	Inadequate screening facilities and equipment, such as pulse oximeters and echocardiogram machines, Lack of dedicated screening areas Insufficiently trained personnel
Training and Knowledge Gaps	Limited training opportunities Lack of updated screening techniques
Workload and Time Constraints	Limited time for screenings Exhaustion and overburdened staff
Emotional and Psychological Burden	Emotional distress in dealing with severe CHD cases Lack of psychological support for healthcare professionals
Parental Unawareness and Consent Issues	Lack of parental knowledge regarding CHD screening Challenges in obtaining informed consent Parental mistrust and reluctance
Socioeconomic Challenges	Financial constraints for screening and follow-up Difficulty in accessing healthcare facilities Low literacy levels impacting healthcare decisions
Operational Challenges	Absence of referral protocols Poor coordination among healthcare teams Inconsistencies in screening practices Lack of an electronic data management system
Implications of Policy and Regulations	Absence of national standardized screening protocols Lack of integration of CHD screening into routine care Limited availability of telemedicine and follow-up services
Suggestions and Recommendations from HPs	Investment in diagnostic tools Continuous training and skill development Implementation of uniform national screening guidelines Awareness campaigns for parents Strengthening referral systems and financial support for families

## Data Availability

All the data are included in this manuscript.

## Supplementary Materials

The following supporting information can be downloaded at: https://www.mdpi.com/article/10.3390/ijns11040095/s1.

## Abbreviations

CHD	Congenital Heart disease
LMICs	Low and middle income countries
HPs	Health professionals
STHS	Saidu Group of Teaching Hospital Swat
SHS	Shifa Hospital Swat
SIMS	Swat Institute of Medical Sciences
