# Supplementary material for: Challenges Faced by Healthcare Professionals in Screening Newborns for Congenital Heart Defects in Pakistan
Source: Int J Neonatal Screen. 2025 Oct 15;11(4):95. doi: 10.3390/ijns11040095 (PMC12551085; doi:10.3390/ijns11040095)
Supplement: Supplementary file 1 [file IJNS-11-00095-s001.zip › Supplementary Materials.pdf]

Supplementary Materials

The questionnaire contains the following questions to help understand the challenges in screening CHD among newborns.

Professional Role of the Participant
Experience in providing care to newborns with CHD
Type of Facility
Location of the facility
Can you explain the screening method for CHD used at your facility?
What are the screening tools utilized at your facility?
Are there any challenges in utilizing screening tools?
How often is echocardiography conducted for early screening such as fetal echo or echo at birth?
Are there any challenges in utilizing Echo?
How do you perceive the effectiveness of current screening methods for CHD in newborns?
How is data regarding CHD screening collected and managed in your facility?
What are the common limitations you face in the screening process?
Are there any regulations or policies affecting the screening of CHD?
Can you discuss any ethical considerations you face in the screening process?
How do you involve caregivers in the screening process, and what challenges do you face in communicating with them?
Are there any cultural practices or social factors in your community influencing CHD screening?
Do you think there are difficulties in the accessibility of screening services, especially for the rural population?
How do you think the screening process can be improved?
How effective is the referral system for newborns suspected or diagnosed to specialized care?
Are there any follow-up (FU) practices for newborns at risk or diagnosed with CHD?
In your opinion, how frequently are parents missing FU appointments or not adhering to prescribed care plans?
In your experience, what local factors (cultural, socioeconomic and geographical) impact the success of FU care for CHD?
Do you face difficulties coordinating FU care with other healthcare providers (e.g., referral systems, communication gaps between specialists and general physicians)?
Are there any successful programs or interventions in other settings that you believe could be adapted here?
Would you require additional training or resources to implement a standardized FUF?
What are your recommendations for improving the FU care for neonates with CHD in Pakistan?
