# Supplementary material for: Challenges Faced by Healthcare Professionals in Screening Newborns for Congenital Heart Defects in Pakistan
Source: Int J Neonatal Screen. 2025 Oct 15;11(4):95. doi: 10.3390/ijns11040095 (PMC12551085; doi:10.3390/ijns11040095)
Supplement: Supplementary file 1 [file IJNS-11-00095-s001.zip › Standards for Reporting Qualitative Research (1).pdf]

Standards for Reporting Qualitative Research

No. Topic	Item
**Title and abstract**	Challenges For Health Professionals in Screening Congenital Heart Defects Among Newborns.
S1 Title	Qualitative interviews with health professionals were conducted to understand the challenges in screening congenital heart defects among newborns.
S2 Abstract	This study explores the challenges faced by health professionals in screening for congenital heart defects (CHDs) among newborns. Using a qualitative approach, the research identifies barriers such as limited training, resource constraints, and diagnostic complexities. Findings highlight the need for improved protocols, training, and support systems to enhance early detection and outcomes.<br><br>**Objectives:**<br>• To understand the challenges of health professionals in screening congenital heart defects in newborns.<br>• To take suggestions from health professionals for improving and extending screening practices.<br><br>**Methods:**<br>A qualitative descriptive research design employed semi-structured interviews to obtain a detailed description of the challenges HPs face in screening CHD among newborns. A purposive sampling technique was used to recruit a diversified sample of 25 HPs from three facilities in district Swat and the principle of information saturation was followed to obtain saturated responses.<br>**Results:**<br>Various challenges highlighted are resource and infrastructure constraints, training and knowledge gaps, workload and time constraints, policy and regulations, emotional and psychological burdens, operational challenges, socioeconomic parental unawareness and consent issues.<br>**Conclusions:**<br>The multifaceted challenges faced by HPs during CHD screening of newborns in resource-limited settings are highlighted and suggestions are given for improvement.
**Introduction**	
S3 Problem formulation	Congenital heart defects (CHD) are one of the most common birth defects worldwide, affecting about 1.35 million children annually with a prevalence of 1%. Screening for early detection is an essential entity practiced worldwide to minimize deteriorating outcomes. The health professionals screening CHD in newborns face several challenges affecting their performance leading to ineffective screening which results in unwanted outcomes such as delay in the detection of CHD increasing morbidity, complications, disability and mortality.

S4 Purpose or research question	This study evaluates the challenges healthcare professionals (HPs) face in screening CHD among newborns. It explores the limitations of current screening practices, disparities in resources, and the need for improved education and training.<br>• To understand the challenges of health professionals in screening congenital heart defects in newborns.<br>• To take suggestions from health professionals for improving and extending screening practices.
**Methods**	
S5 Qualitative approach and research paradigm	The lived experiences of health professionals involved in CHD screening among newborns were explored using a phenomenological approach. This study was grounded in a constructivist paradigm and emphasized the subjective nature of the challenges.
S6 Researcher characteristics and reflexivity	The research team included a variety of experts involved in pediatric cardiac care and qualitative research. Regular meetings and discussion on findings acknowledge biases and assumptions.
S7 Context	The study was conducted in Saidu Teaching Hospital Swat, Khyber Pakhtunkhwa Pakistan, a tertiary care facility providing CHD screening services.
S8 Sampling strategy	Health Professionals such as pediatric cardiologists, pediatricians, cardiologists, nurses and allied health professionals with 1 year experience in screening CHD among newborns were purposively selected to construct a sample of 25.
S9 Ethical issues pertaining to human subjects	Ethical approval was obtained from the institutional review board of Saidu Teaching Hospital/ Saidu Medical College Swat. Consent from each participant was obtained ensuring confidentiality and security of their data.
S10 Data collection methods	Semi-structured interviews with health professionals were conducted to gather in-depth insights into the challenges faced by them during screening CHD in newborns.
S11 Data collection instruments and technologies	Interviews were recorded both in writing and audio records. The data was managed according to the Lincoln and Guba criteria for credibility, dependability, confirmability, and transferability.
S12 Units of study	Health professionals working at different positions and involved in CHD screening among newborns for at least one year.
S13 Data processing	The data was processed in an anonymized way, interview transcripts were kept confidential and coded thematically.
S14 Data analysis	Data analysis followed the thematic analysis approach outlined by Braun and Clarke 2006 to identify recurring patterns and themes related to screening challenges.
S15 Techniques to Enhance Trustworthiness	The trustworthiness was ensured by member checking, triangulation and maintaining an audit trail.
**Results/Findings**	
S16 Synthesis and interpretation	The main themes emerged are: (1) Resource constraints, including lack of equipment and personnel (2) Limited training of health professionals (3) Diagnostic complexities due to subtle clinical presentations of CHDs.
S17 Links to empirical data	Each theme was illustrated by direct quotes from participants, providing empirical support for the findings

**Discussion**	
S18 Integration with prior work, implications, transferability, and contribution(s) to the field	The findings of this study coincide with previous research studies highlighting systemic challenges in CHD screening. A need for targeted training programs, resource allocation, standardized screening protocols and socioeconomic support for families are suggested for improving screening. These insights can be transferred to similar healthcare settings on a wider scale.
S19 Limitations	The possible limitations are response bias, as responses of HPs may be influenced by recall bias, social desirability and reluctance to disclose challenges related to institutional constraints.
**Other**	
S20 Conflicts of interest	The authors declare no conflicts of interest.
S21 Funding	The conduction of this study involves no funding or support source for data collection, interpretation and reporting of the findings.
